# The HIV-1 Vpr Protein: A Multifaceted Target for Therapeutic Intervention

**DOI:** 10.3390/ijms18010126

**Published:** 2017-01-10

**Authors:** María Eugenia González

**Affiliations:** Unidad de Expresión Viral, Centro Nacional de Microbiología, Instituto de Salud Carlos III, Carretera de Majadahonda-Pozuelo Km 2, Majadahonda, 28220 Madrid, Spain; megonzalez@isciii.es; Tel.: +34-91-822-3698; Fax: +34-91-509-7966

**Keywords:** Vpr protein, mechanism of action, pathogenesis, cellular metabolism, antiretroviral target

## Abstract

The human immunodeficiency virus type 1 (HIV-1) Vpr protein is an attractive target for antiretroviral drug development. The conservation both of the structure along virus evolution and the amino acid sequence in viral isolates from patients underlines the importance of Vpr for the establishment and progression of HIV-1 disease. While its contribution to virus replication in dividing and non-dividing cells and to the pathogenesis of HIV-1 in many different cell types, both extracellular and intracellular forms, have been extensively studied, its precise mechanism of action nevertheless remains enigmatic. The present review discusses how the apparently multifaceted interplay between Vpr and host cells may be due to the impairment of basic metabolic pathways. Vpr protein modifies host cell energy metabolism, oxidative status, and proteasome function, all of which are likely conditioned by the concentration and multimerization of the protein. The characterization of Vpr domains along with new laboratory tools for the assessment of their function has become increasingly relevant in recent years. With these advances, it is conceivable that drug discovery efforts involving Vpr-targeted antiretrovirals will experience substantial growth in the coming years.

## 1. Introduction

The Vpr protein encoded by the human Immunodeficiency virus type 1 (HIV-1), formerly named Viral Protein R, is associated with replication efficiency and cytopathogenicity of the virus [1,2]. Early studies suggested that this 96-amino acid protein was dispensable in vitro for HIV-1 replication and cytopathogenicity in lymphoid cells, and for that reason was considered an “accessory” protein [3]. However, later studies demonstrated the important role that Vpr plays in vivo for virus spread and pathogenesis [4]. Along this line, several studies demonstrated that Vpr increases the rate of viral replication and accelerates the cytopathic effect of the virus on T cells and, more importantly, is essential for viral replication in macrophages [5,6,7]. More detailed analysis clarified that while Vpr was important for virus replication in primary monocytes and differentiated macrophages, it was superfluous for the infection of resting or stimulated peripheral blood mononuclear cells or CD4^+^ cell lines [8,9]. Follow-up research indicated that Vpr promotes early T cell activation, facilitating productive HIV-1 infection of non-activated T cells [10]. Significant amounts of Vpr protein and anti-Vpr antibodies can be detected in the serum of HIV-1-infected patients [2,4]. Serum Vpr has been linked to the activation of HIV-1 replication in vivo and also with the control of latency [4]. The observed injury in the central nervous system (CNS) of certain HIV infected patients might be related to the neuronal cell death caused by Vpr protein [11,12]. It is important to highlight that Vpr is one of the HIV-1 virion-associated proteins, reaching more than 700 molecules per virion; approximately 1:7 compared with the 5000 Gag molecules estimated by cryo-electron microscopy (EM) and scanning transmission EM [13,14,15]. Once the virion enters the cell, the Vpr protein shuttles between cytoplasm and nucleus to facilitate viral replication and, at late stages in the virus life cycle, newly synthesized Vpr is exported from the cell [16,17]. Two forms of nuclear Vpr have been observed, monomers and very large complexes, 1000× larger than a monomer [17]. Thus, Vpr has been considered as a vehicle to deliver antiviral molecules into virions and also as a potential therapeutic target to block HIV disease [18,19]. Furthermore, the fact that all primate lentiviruses share a *vpr* gene whose translation product shows highly conserved motifs along the amino acid sequence highlights its fundamental role in primate lentivirus evolution and biology [20,21]. Therefore, the proposed essential role of Vpr for in vivo viral infection and for disease progression is now beyond doubt [9,22].

The absence of an HIV vaccine and the unlikely prospect of its availability in the foreseeable future necessitates the development of an arsenal of anti-HIV drugs to control and treat the infectious disease. Notwithstanding the significant progress achieved, available antiretroviral strategies are not capable of eradicating HIV in treated patients due to viral reservoirs within cells and tissues, emergence of resistant viruses and adverse effects associated with each antiviral drug class. The challenges of improving efficacy and reducing toxicity of current highly active antiretroviral therapy (HAART) demands the exploration of novel targets to develop new drugs to add to those already in use [23]. This review aims to provide a comprehensive update on the state of the art of the multiple molecular mechanisms of HIV-1 Vpr that makes it an attractive candidate for antiretroviral therapy.

## 2. Origin and Conservation of Vpr Protein from Human Lentiviruses

**Figure 1:**
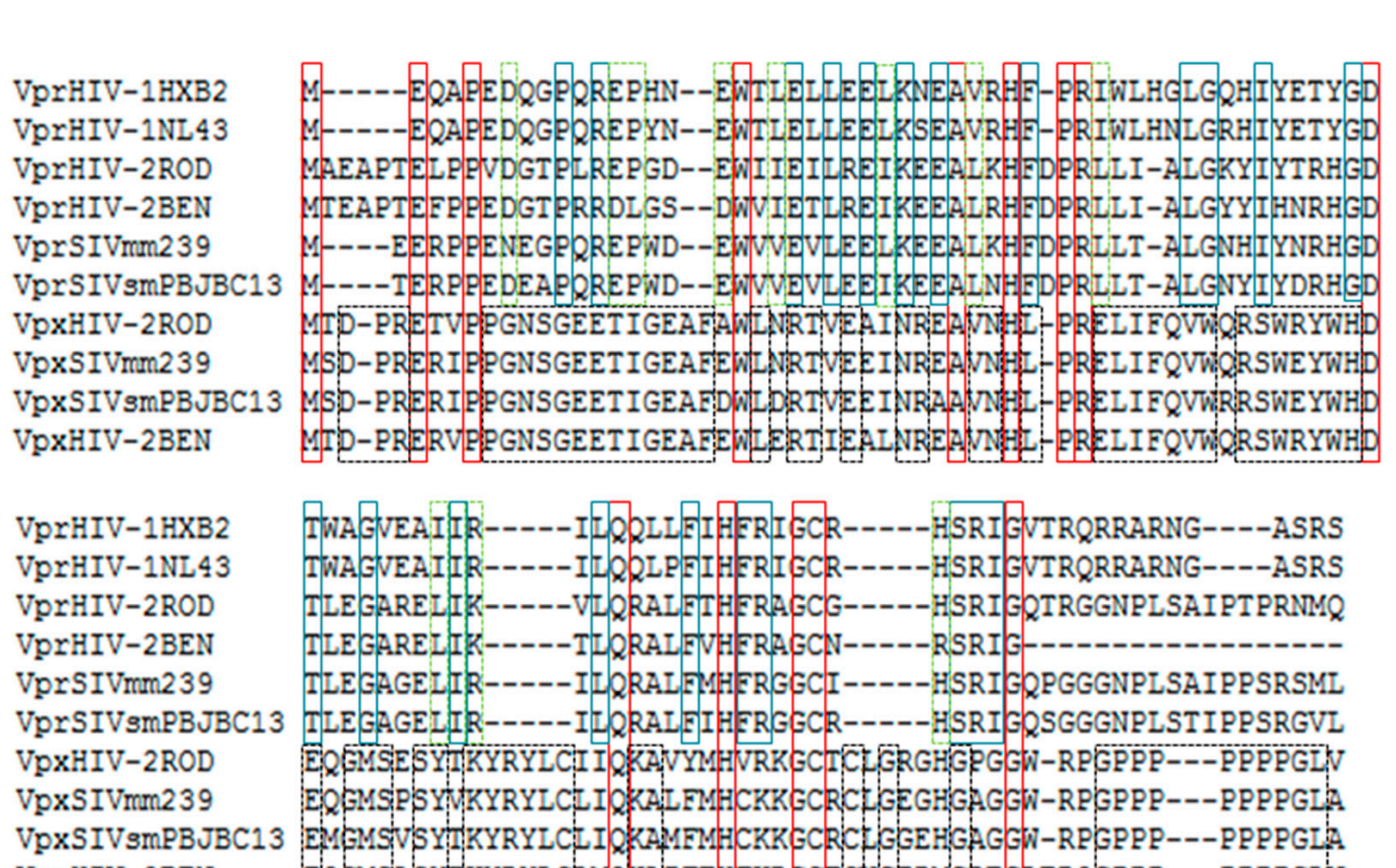
Amino acid sequence alignment of Vpr and Vpx proteins from human Immunodeficiency virus type 1 (HIV-1) and type 2 (HIV-2), and simian immunodeficiency viruses (SIV) using Fast Fourier Transform (MAFFT) at the EMBL-EBI server (available on 20 October 2016 http://www.ebi.ac.uk). Color code of amino acids within rectangles: red = fully conserved residues in all Vpr and Vpx proteins; blue = fully conserved residues only in Vpr proteins; dotted green = residues with similar properties in Vpr proteins; dotted black = fully conserved residues only in Vpx proteins.

Although HIV-1 *vpr* is selected against during the establishment of persistent infection in tissue culture, there is a positive selection for Vpr function in vivo in HIV-1 and simian immunodeficiency virus SIV_CPZ_ [24,25]. All primate lentiviruses contain a *vpr* gene in their genome. HIV-2 and SIV_SM_ genomes additionally carry the *vpx* gene, which is similar to the unique *vpr* of SIV_AGM_ [26]. Despite this apparent redundancy, both genes have evolved in HIV-2/SIV_SM_ to produce Vpr and Vpx proteins and each one executes only one of the two functions that are together developed by Vpr HIV-1/SIV_CPZ_ and Vpr SIV_AGM_ proteins [27]. Several residue changes were incorporated into both proteins to be able to interact with cellular factors of new host species, although the similarity in their overall structure is preserved [20,28]. Actually, there is low identity of amino acids between Vpr and Vpx sequences (Figure 1). The structure of HIV-1 Vpr protein is characterized by three well-defined α-helices at amino acid positions, 17–33, 38–50, and 54–77, surrounded by flexible N- and C-terminal domains [29]. Vpr and Vpx proteins from HIV-2 also have three amphipatic α-helices [30] and amino acid identities among the three proteins are particularly concentrated at helix 1 and helix 3.

Viruses need to counteract intrinsic antiviral factors that restrict viral replication in host cells and for this purpose HIV and SIV might have acquired accessory proteins along their evolution [31]. The comparison of the antagonism patterns between Vpr, Vpx, and another accessory protein, Vif, from primate lentiviruses toward host antiviral factors such as SAMHD1 and APOBEC3 proteins suggests that the loss of the *vpx* gene and adaptation to hominids, by reconstruction of the overlapping *vif* gene, occurred during the passage of SIVs from old world monkeys through chimpanzees [32], subsequently facilitating the adaptation of HIV-1 to humans. Thus, the conservation of Vpr functions together with the genomic adaptations to antagonize the antiviral factors of new host species could have determined the evolution of primate lentiviruses [33].

## 3. The Role of Vpr Protein in HIV-1 Infection and Disease Progression

One of the outstanding activities performed by Vpr after HIV-1 infection of dividing cells is the blockade of the cell cycle at G2 [25,34,35,36], a phase where the viral long terminal repeat (LTR) promoter is more active [24]. Equally relevant is its activity in non-dividing cells, where its contribution to the nuclear import of the viral preintegration complex (PIC) is critical for virus replication in these cells [9,37]. Other detected activities for Vpr include the regulation of apoptosis and the transcriptional modulation of immune function [38,39,40,41,42]. The relevance of these activities for infection competence is not clear and becomes even more complex when one considers the following issues that could be crucial for in vitro-in vivo extrapolation. Vpr is able to form dimers and even multimers that may determine its functions [42]. While only oligomerizable Vpr incorporates into virus particles and has nuclear transport ability, non-oligomerizable variants retain some activities of the protein, such as inhibition of cellular proliferation and also bystander cell death [42,43,44]. The multiple localization of Vpr protein in infected cells, including inside the nucleus, in mitochondria and dispersed in the cytoplasm, could account for its diverse functions [45,46,47]. It is conceivable that the origin of the protein, released from virions or de novo synthesized, will determine which of the diverse range of activities is prominent when Vpr interacts with each cell type [48,49]. Moreover, differences in the level of its expression might explain the timing of Vpr functionality along the viral replication cycle [50]. The variety of molecular events leading to innate recognition of HIV in different target cells, low permissiveness to infection of primary cells, and defects of cell lines in the release of cytokines to the extracellular milieu are key factors to bear in mind when attempting in vitro assessment of the Vpr modulation of antiviral immune response [51].

### 3.1. Concurrence and Independence of Cytopathogenic Activities of Vpr

The links between the activities described for Vpr protein have been extensively investigated. The two principal features of HIV-1 Vpr, cell cycle arrest and promotion of macrophage infection, seem to be independent since as shown in other lentiviruses, SIV_SM_ and HIV-2, they are segregated into two proteins, Vpr and Vpx [27,52,53,54]. Nevertheless, several cause-effect relationships have been suggested, especially relating to Vpr-induced G2 arrest with other activities. For example, entry into G2 was proposed to be required for Vpr to induce apoptosis [55]. However, the independence of both Vpr activities was demonstrated with Vpr mutants that do not arrest the cell cycle but retain cell death activity, and vice versa [43,56]. Moreover, results from live imaging suggested that Vpr induces apoptosis in G1 and M phase but fails to induce significant cell death in S or G2 phases [57]. Additionally, the increased expression of HIV-1 LTR in G2-arrested cells might be explained by the cell cycle regulation of some transcription factors [24,58]. Other likely links were proposed for the Vpr-induced nuclear import of the HIV-1 PIC with G2 arrest in CD4^+^ T cells or with stimulation of virus replication in macrophages [59,60,61]. Conversely, the analysis of Vpr mutants suggested that nuclear import, virion incorporation, and cell cycle arrest/differentiation are mediated by distinct functional domains of HIV-1 Vpr [62]. Nonetheless, later findings associated Vpr activities with Vpr interference with the proteasome pathway by interacting with DNA Damage-binding protein 1 (DDB1)-and-Cullin-4-associated Factor 1 (DCAF1) [28]. Thus, the molecular mechanisms of Vpr-induced cell cycle arrest, cell death, nuclear import, and virus activation are still the subject of study. The latest advances in the understanding of Vpr function indicate that some Vpr activities are likely related to the functioning of the cellular machinery and host factors, as will be discussed below.

### 3.2. The Influence of Vpr on Disease Progression

Vpr protein likely contributes to disease progression in HIV-1-infected patients in several ways: (1) by inhibiting the proliferation of T cells and inducing cellular differentiation [20,31,63]; (2) by enabling productive infection of primary macrophages and reactivating virus production from latency, which contributes to virus production in the absence of CD4^+^ T cells and to the establishment of drug resistant reservoirs in patients early in infection [4,6,7,18,64]; (3) by contributing to the bystander cell depletion in lymphoid tissues, peripheral blood, and the CNS [12,38,65]. In this respect, soluble Vpr can activate cells in an autocrine or paracrine manner, and this activation could contribute to immune deficiency in patients [4]. It has been proposed that Vpr intervention enables HIV to circumvent the innate immune sensing of viral infection and to prevent the triggering of an innate immune response [51,66]. However, this notion is currently under debate; a recent study has reported the Vpr-mediated potentiation of HIV-1 sensing in CD4^+^ T [41]. All of this without forgetting that Vpr can traverse the blood brain barrier as an extracellular soluble protein or as an intracellular protein in lymphocytes and monocytes-macrophages. Additionally, infected brain microvascular endothelial cells and brain resident cells might also release soluble Vpr in the CNS. Once there, extracellular Vpr might directly contribute to the HIV-associated CNS dysfunction or through bystander effects mediated by factors involved in cellular death pathways [46,67].

In macrophages, HIV-1 productive infection is low and infected-cells survive and become viral reservoirs [35,68]. It is proposed that Vpr induces anti-apoptotic pathways in infected macrophages that facilitate viral replication and long-term cell survival. Furthermore, a recent study has shown that, by altering the maturation of their phagosomes Vpr impairs the phagocytic function of macrophages, which in turn could contribute to the establishment of opportunistic infections in HIV-infected patients [69,70].

## 4. Functional Perturbation of Cells by the HIV-1 Vpr Protein

Vpr protein contributes to the pathogenesis of HIV-1 by direct and indirect disruption of cellular homeostatic mechanisms. In the context of viral infection or on its own, Vpr triggers cell death in several cell types [12,16,38,71,72,73]. Consequently, Vpr is associated with the induction of immune activation by depleting regulatory CD4^+^ T cells with subsequent massive propagation of CCR5-tropic HIV-1 in vivo [74]. Similarly, by activating Nuclear Factor of Activated T cells (NFAT) in primary T cells, Vpr primes them for productive infection [10]. Moreover, Vpr promotes the release of tumor necrosis factor-alpha by infected lymphocytes [75], and the secretion of this proinflammatory cytokine is associated with rapid disease progression [76,77,78]. Additionally, Vpr induces the expression of other cytokines involved in the regulation of inflammation, such as IL-8, and in the recruitment of immune cells, such as IL-6 [79,80]. By contrast, IL-12, a critical Th1 cytokine secreted by antigen presenting cells, is suppressed by Vpr [81].

It has been demonstrated that purified extracellular Vpr can enter cells, suggesting that circulating Vpr could affect bystander cells in infected patients [18,76]. Extracellular Vpr permeabilizes the cell membrane to calcium and magnesium [82]. Moreover, as a result of Vpr-induced permeabilization of the permeability transition pore complex (PTPC), apoptogenic factors are released from the mitochondrial membrane [83]. Vpr-induced deregulation of genes involved in calcium homeostasis is related to neuronal deregulation and loss [84]. In yeast, Vpr-induced mitochondrial dysfunction transiently produces respiratory deficiency and cells are unable to utilize ethanol or glycerol as the sole carbon source [85]. In addition, cell death induced by Vpr is occasionally independent of caspases, having the hallmarks of necrosis [86].

The molecular dissection of Vpr interactions with cells has been possible through the analysis of cells infected with viruses carrying Vpr variants, Vpr-transfected cells, and from the use of Vpr-derived peptides or *vpr*-antisense ribonucleotides for expression or inhibition studies, respectively. This, together with various cellular models including yeast, primary cells, established cell lines, and also animal models, has permitted the analysis of the specific cellular mechanisms which are modified by Vpr [82,85,87,88,89,90,91]. Moreover, proteomic analysis of HIV-1 transgenic rat models has identified modifications in the content of cellular proteins involved in these Vpr-targeted pathways [92,93].

## 5. Molecular Mechanisms That Are Affected by Vpr Interaction with Cellular Factors

Several proteomic analyses have been undertaken in an attempt to unravel the precise mechanism of action of HIV-1 Vpr. Data obtained from these studies revealed Vpr-induced changes to protein mediators and modulators of signaling pathways related to glycolysis and other energy processes, mitochondrial activity, redox homeostasis, cell cycle, cell death, and DNA repair [93,94,95,96]. It is also likely that the mitochondrial dysfunction provoked by Vpr affects proteasomal activity [93,97], which would further impact the regulation of transcription initiation [98]. In some cases there appear to be causal links between the effects of Vpr in host cells, undoubtedly because of the variety of signaling pathways affected by viral protein. In addition to transcription and translation, the turn-over of cellular proteins regulates cellular processes. This section is intended to contextualize the significant changes in the amount/activity of some cellular proteins by Vpr (Table 1).

### 5.1. Energy Pathways, Redox Homeostasis, and Cell Cycle

Molecular biological analyses in a wide variety of virus families suggest that virus production requires glycolysis during later steps in replication [99]. HIV-1 infection of T cells increases glycolysis, whereas infection of macrophages suppresses glycolysis [100]. This cell type-dependent adaptation of glucose metabolism agrees well with the known differences in virus production and cell survival in both cell types. In macrophages, *vpr* transduction enhances the expression of glucose-6-phosphate dehydrogenase (G6PDH), a pentose phosphate pathway (PPP) enzyme that functions as a sentinel for oxidative stress, while it reduces the expression of glyceraldehyde 3-phosphate dehydrogenase (GAPDH), a key glycolytic enzyme [94]. Decreased GAPDH activity by extracellular Vpr is also observed in astrocytes [65].

Besides GAPDH, several key mitochondrial enzymes involved in glutamate metabolism are significantly downregulated by Vpr in macrophages, among them glutamate dehydrogenase 2 (GLUD2), which may contribute to neuronal pathogenesis [94]. Conversely, the survival of HIV-1-infected macrophages has been related to the capacity of Vpr to preserve mitochondrial integrity by increasing the amount of mitochondria-bound hexokinase (HK-1) [101]. In yeast, Vpr triggers the loss of respiratory chain complex I–IV and citrate synthase activities [85]. These data suggest that the G2 growth arrest may be an epiphenomenon of Vpr-induced mitochondrial dysfunction that causes loss of the mitochondrial respiratory function with subsequent ATP deficiency. This suggestion is in agreement with the recent demonstration that glucose restriction provokes transient G2 arrest of yeast [102]. HIV-1-infected individuals display multiple symptoms of redox imbalance and Vpr is one of the viral proteins thought to be involved in these phenomena [56,103,104,105]. This hypothesis was later proven using different experimental approaches. Early studies demonstrated that drugs that replenish intracellular glutathione GSH also counteracted oxidative stress and inhibited HIV replication in models of acute and latent infection [106]. Specific assays using extracellular Vpr protein demonstrated impaired levels of intracellular ATP and GSH in astrocytes [107]. Furthermore, extracellular addition of ATP or GSH and its precursors was sufficient to counter growth arrest by endogenously-produced Vpr in yeasts [108]. These treatments also ameliorated Vpr-induced growth impairment in respiratory-deficient (*petite*) mutants of *Saccharomyces cerevisiae*, pointing to a Vpr effect in an energy pathway different than oxidative phosphorylation.

Whereas physiological concentrations of endogenous ATP downregulates proteasome activity, it is rapidly upregulated by reduced ATP [109]. Thus, a Vpr-induced reduction in intracellular ATP might explain the observed decrease of endogenous cellular proteins. HIV-1 infection decreases the abundance of mitochondrial ion channels (VDAC1, VDAC2) and glutathione reductase (GSR), which respectively facilitates the survival of infected cells and protects late stages of virus production [110,111]. Consequently, GSH might exert an antiviral effect by impairing the proper assembly of virus proteins [112]. It should be noted that the functional interplay of peroxisomes with other subcellular organelles, such as mitochondria, is necessary for the regulation of cellular redox metabolism [113].

Hypoxia inducible factor-1 alpha (HIF-1α) has been proposed as the major transcription factor participating in the Vpr-mediated activation of the HIV-1 promoter [114]. The activation of this transcription factor would occur once Vpr activates the oxidative stress pathway [115]. Supporting this model is the finding that the switch from HIV-1 latency to reactivation in infected macrophages is promoted by a marginal increase in glutathione redox potential (E_GSH_) of about 25 mV [116]. A moderate oxidative shift in E_GSH_, a consequence of GSH oxidation to glutathione disulfide (GSSG), is detected at early stages of viral replication, but as viral replication increases, higher oxidation and also depletion of GSH leads to a robust oxidative shift in E_GSH_. Indeed, chronic HIV-1 infection enables a better redox response and tolerance to apoptosis.

Early studies associated the Vpr-induced accumulation of cells in G2 phase with the accumulation of the hyperphosphorylated form of the cyclin-dependent kinase cell division control 2 (CDC2) [34,35]. Consequently, Vpr would prevent the required activation of p34*^cdc2^*/cyclin B complex for entry into M phase. Later studies found that Vpr activates the stress-induced kinase ataxia telangiectaxia and Rad3-related (ATR), which results in phosphorylation of various substrates including Chk1 [117]. While previous evidence suggested that Vpr-induced G2 arrest does not use classical checkpoint pathways [118,119], subsequent studies demonstrated that Vpr directly binds to chromatin and the ATR stress signaling pathway then becomes activated [120]. The mechanism by which Vpr protein activates this DNA repair response is, however, not clear since ATR responds to a broad spectrum of DNA damage [121]. Alternative models to explain Vpr-mediated G2 arrest rely on the proteasome-mediated downregulation of several cellular factors. Among them are the structure-specific endonuclease regulator SLX4, histone deacetylases (HDAC), the DNA replication factor minichromosome maintenance 10 (MCM10), and also unknown factors [66,122,123,124,125,126].

### 5.2. Proteasomal Activity and Cell Death

The identification of an endogenous cellular protein that binds to Vpr, VprBP/DCAF1, provided a crucial clue to elucidate the molecular mechanisms of Vpr-induced cell cycle arrest [127,128]. The discovery of the interaction between Vpr and DCAF1 gave rise to alternative models to explain the potential depletion of cellular factors that could be required for cell cycle progression [123,124,125,126,129,130]. DCAF1 is an element of the E3 ubiquitin ligase complex. The DCAF1-DDB1-Cul4 E3 ubiquitin ligase complex is involved in the facilitation of macrophage infection and Vpr-mediated protein degradation. Thus, the hijacking of host DCAF1-CUL4 E3 ubiquitin ligase by Vpr enables targeting of the endonuclease complex MUS81 structure specific endonuclease subunit/essential meiotic structure-specific endonuclease 1 (MUS81/EME1) for degradation via the proteasome and also the activation of SLX4 endonuclease complex that promotes G2/M arrest and escape from innate immune sensing [66,131]. In addition, Vpr-induced acceleration of DCAF1 turnover protects viral envelope (Env) protein from lysosomal degradation and enhances virion production in macrophages [132]. Indeed, Vpr and DCAF1 were found to be necessary for efficient cell-to-cell spread of HIV-1 from macrophages to CD4^+^ T lymphocytes [133]. The Vpr-induced mitochondrial dysfunction might affect proteasomal activity and vice versa [109]. Ubiquitin-proteasome system and mitochondria are involved in the cellular response to oxidative stress and intracellular variation of ATP levels. Several authors have proposed that Vpr has a dual pro-apoptotic or anti-apoptotic role on programmed cell death that is dependent on its intracellular level, the stage of the infection, and also the cell type [39,50,133,134]. Vpr permeabilizes mitochondrial membranes through a specific interaction with the PTPC via interaction with the adenine nucleotide translocator (ANT) that is located in the inner mitochondrial membrane [83]. This interaction results in the permeabilization of the inner mitochondrial membrane. The subsequent swelling of the mitochondrial matrix might result in impairment of the outer mitochondrial membrane [135,136]. Thus, it has been proposed that Vpr induces a mitochondria-dependent apoptotic pathway in T cells and primary mononuclear cells [137]. In neurons, an alternative mechanism has been proposed where the uptake of extracellular Vpr permeabilizes the plasma membrane by downregulating the plasma membrane Ca^2+^ ATPase (PMCA) [138]. As a consequence, Vpr triggers an increase of intracellular Ca^2+^ levels, leading to ROS production and impairing signaling in neurons. Conversely, Vpr can regulate in transfected T cells the oncogene bcl-2, which is endowed with anti-apoptotic activity and downmodulates the pro-apoptotic factor bax, protecting the mitochondrial outer membrane from permeabilization [139]. In this manner, and at early stages of infection, low levels of endogenous Vpr may protect T lymphocytes from death, contributing to the virus dispersion. In addition, the alternative anti-apoptotic role that ATR plays in the mitochondria might contribute to the regulation of apoptosis by Vpr [140].

### 5.3. Transcriptional Regulation

Early observations revealed that extracellular Vpr was capable of reactivating HIV-1 virus from latency [4,18]. Direct interaction of Vpr with the glucocorticoid receptor (GR) and/or other components of the glucocorticoid-induced transcription initiation complex would signal the transactivation of HIV LTR [141,142]. Further investigation showed that this was due to its ability to transactivate several promoters, among them the viral LTR [5,143,144,145]. According to a recent study, this transactivation capacity occurs via the depletion of histone deacetylase (HDAC) 1 by Vpr. In T cells Vpr increases the basal ubiquitination of HDAC1 and HDAC3 by 2.2- and 3.4-fold, respectively [122]. In infected macrophages, Vpr induced depletion of HDAC1 on specific chromatin regions was associated with hyperacetylation of histones and consequently the activation of the viral promoter [126]. Hence, Vpr may enable the virus to overcome latent infection in primary macrophages. In resting CD4^+^ cells, virion encapsidated Vpr activates NFAT through Ca^2+^ influx and the nuclear import of this transcription factor [10]. Modification of the regulation of several transcription factors, such as NF-κB, AP-1, and C/EBP-delta by Vpr could be the cause of the observed Vpr–induced impairment of cytokines and GR signaling in a broad range of cell types [79,80,81].

### 5.4. DNA Repair Mechanisms

Curiously, the HIV-1 Vpr directs two repair enzymes, helicase-like transcription factor (HLTF) and uracil DNA glycosylase (UNG2), for proteasome-dependent degradation, while the HIV-2 Vpr targets the dNTPase SAM and HD domain containing deoxynucleoside triphosphate triphosphohydrolase 1 (SAMHD1) [53]. Thus, both Vpr proteins reprogram CRL4 (DCAF1) E3 ligase to remove key enzymes involved in three DNA repair pathways, although each protein uses a different strategy to achieve this [53]. HIV-1 Vpr interacts with UNG2, a nuclear DNA repair enzyme that excises uracil from DNA containing miss-incorporated deoxyuridine triphosphate (dUTP), leading to its degradation [146,147,148]. The association of Vpr with the DDB1-containing E3 ligase complex mediates the degradation of UNG2 [149]. The relevance of UNG2 counteraction by Vpr is controversial. On the one hand, Vpr recruits UNG2 into HIV-1 particles in producer cells and consequently modulates virus mutation rate [88,90]. On the other hand, retroviral DNA uraciliation requires exceptional conditions in the host cell in order to be relevant as a barrier to HIV-1 infection. These requirements are low dUTPase levels, high dUTP levels, and abundant nuclear hUNG2; however, not all of these conditions coincide in macrophages or CD4^+^ T cells [150]. Concerning the HIV-1 Vpr-mediated downregulation of the DNA translocase, HLTF, a recent study demonstrated that this occurs independently of cell cycle stage [96]. As far as is known, the depletion of this DNA translocase occurs in a DCAF1-dependent manner in T cells and macrophages.

## 6. Amino Acid Residues Contributing to Vpr Functions

**Figure 2:**
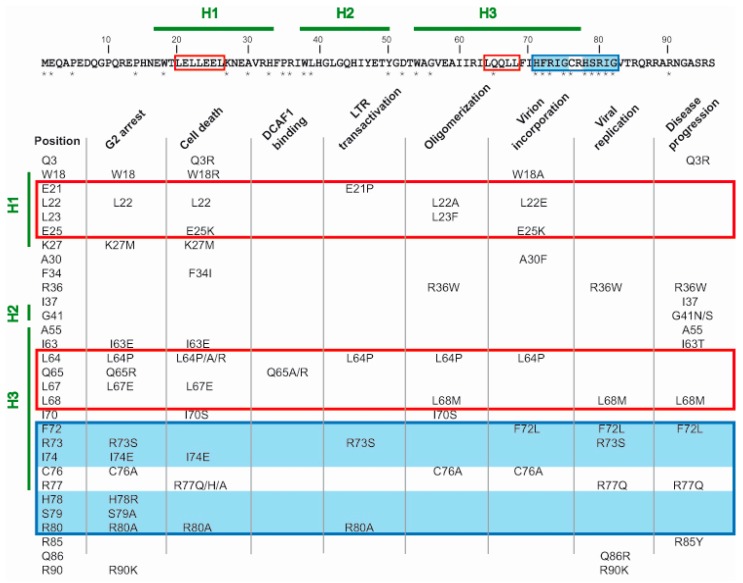
Summary of single sequence polymorphisms that have been associated with alteration in any of the activities of Vpr. The HXB2 Vpr represents the consensus sequence. * symbols indicate positions which have fully conserved residues in HIV-1 Vpr isolates from different M subtypes. H1, H2, and H3 refer to the three α-helices. Relevant residues are outlined in rectangles with the following color code: red = leucine-rich domain, blue = mitochondrial membrane permeabilization (MMP)-inducing sequence. The two HF/SRIG motifs are shadowed in blue.

The comparison of amino acid sequence between Vpr proteins from different HIV-1 subtypes reveals a high degree of conservation [151]. Several approaches have been undertaken to determine the relationships between sequence amino acid positions and functionality of Vpr. Analysis of Vpr from cultured and natural HIV-1 variants together with site-directed mutagenesis studies have suggested specific domains and residues in the protein sequence that are associated with virus cytopathogenicity and with disease progression (Figure 2).

The *N*-terminal 42 amino acids of Vpr constitute the oligomerization domain of the protein [42]. This domain includes helix 1 and several residues including Q3, W18, L22, L23, K27, and F34, which have been associated with cytopathicity functions of Vpr [10,56,87,152,153,154]. The *C*-terminal moiety (Vpr 52–96) binds to ANT and can induce apoptosis [135]. Two highly conserved leucine-rich domains are located within helix 1 (20–26) and helix 3 (64–68). The first domain is likely involved in the interactions of multimerization and, as a result, virion incorporation, while the second domain binds heterologous proteins such as DCAF1 and GR, which then become coactivated [127,132,155,156]. Non-conservative mutations of L64 enhance the pro-apoptotic activity of Vpr, but in a subtype-dependent manner [157]. Helix 3 contains several hydrophobic amino acids including I63, L67, I70, and I74 that enable nuclear localization, cell cycle arrest, and oligomerization of the viral protein [43]. The protein folds around a hydrophobic core defined by leucine, isoleucine, valine, and aromatic residues located in helix 1, 2, and 3 [29]. The mitochondrial membrane permeabilization-inducing activity of Vpr (MMP) resides within a 12-amino acid moiety (71–82) [135]. This moiety contains two H(F/S)RIG motifs (at 71–75 and 78–82); the conservation of these two motifs correlates with HIV pathogenicity [158]. Located between the two motifs is a very well conserved cysteine residue (C76), which is critical for oligomerization and incorporation into HIV-1 virions, while the H(F/S)RIG motifs are necessary for G2 arrest and/or cell death [10,87,154,158,159,160,161]. Additionally, arginine residues R73, R77, R80, and R90 are strongly conserved, and their mutation reduces virus replication and Vpr-induced activities such as apoptosis, LTR activation, IL-12 suppression, and cell cycle arrest [10,87,130,162,163,164,165,166]. The phosphorylation of S79, but not of S94, and S96, is crucial for the cell cycle arrest, although all three serines can be phosphorylated [154]. Mutations at positions 3, 36, 37, 41, 55, 63, 68, 72, and 77 have been associated with variations in the disease progression or the degree of neurocognitive deficit in patients. The simultaneous presence of A55 and T63 in patient-derived Vpr sequences has been associated with lower plasma viral load and higher CD4 count compared with those that express either single or none of these residues [167]. The impact on the neurocognitive function of patients has been associated with the presence of G41N and A55 (detrimental effect) or I37 and S41 (beneficial effect) [168]. Mutations that have been associated with long-term non-progressor (LTNP) patients are Q3R, Q65R, F72L, and R77Q [153,161,162,163,169]. The R77Q mutation is less frequent among patients with progressive disease (36%) than in LTNP patients (about 80%) and shows poor replication [162,163,170]. Nevertheless, the use of this mutation as a marker of slow disease progression is in disagreement with the lack of correlation of this mutation with the course of disease in progressor patients that were receiving therapy [171,172]. Conversely, rapid progression of disease has been associated with R36W, L68M, and R85Y [162,173].

That different Vpr variants emerge during in vitro infection and different Vpr subtypes from isolates show different pro-apoptotic potential might explain the diversity of HIV-1 pathogenesis [157]. Indeed, the conservation of functional domains and low heterogeneity of the *vpr* gene in mother-infant pair isolates suggests the important role played by Vpr in vertical transmission [174]. Curiously, among the subtypes of HIV-1 Vpr, the subtype C consensus sequence does not function in apoptosis induction as effectively as that from subtype B, but some natural C variants are able to induce cell cycle arrest and apoptosis at a similar rate to subtype B [175]. These observations would support the need for caution when choosing the HIV-1 clone as a reference sequence in drug discovery studies, in order to ensure potential effectiveness of any Vpr-targeted inhibitor in developing countries. Moreover, in some cases single Vpr mutations that are associated with rapid disease progression, do not correlate with the expected Vpr functioning profile [162]. Thus, it is possible that several residues in the Vpr sequence and even Vpr itself, as well as other HIV-1 proteins, make a concomitant contribution to the disease progression.

## 7. Progress in Searching for Vpr-Targeted Drugs

The so-called group of accessory proteins from HIV-1, which includes Nef, Vif, Vpu, and Vpr, has long been considered a promising target for developing therapeutic strategies against acquired immunodeficiency syndrome (AIDS) [176]. The development of effective antiretroviral drugs specifically targeted to Nef, Vif, or Vpu proteins has been attempted using in vitro-selected compounds, although none of these have yet progressed to final approval [177,178,179,180]. Among the accessory proteins, Vpr ranks second only to Nef in terms of the attention generated to halt virus spread. Notably, Vpr packaged into naturally non-infectious virions or into virions deactivated by reverse transcriptase or protease inhibitors retains the capacity of cell cycle arrest [181]. Its determinant role in acutely and latently infected macrophages distinguishes Vpr for HIV eradication by antiretroviral therapy [182,183]. Indeed, soon after the first description of Vpr protein it was shown that antisense phosphorothioate oligodeoxynucleotides complementary to the *vpr* mRNA could inhibit HIV-1 replication in primary human macrophages [6]. Attempts to exploit Vpr activity during viral replication for the development of antiretroviral strategies are many and varied. Early studies explored this protein as a vehicle to target foreign proteins to HIV virions given its packaging into virion particle [19,184]. Investigations on targeting other Vpr activities showed that methylxanthines, such as pentoxifylline and caffeine, inhibited Vpr-induced cell cycle arrest but not Vpr-induced apoptosis [72,185]. Several natural products have also been screened for anti-Vpr activity. As a result, the anthraquinone damnacanthal from the root of *Morinda citrifolia* was reported as an inhibitor of Vpr-induced cell death [186]. Furthermore, several isopimarane diterpenoids extracted from the medicinal herb *Kaempferia pulchra* were recently reported to exhibit potent anti-Vpr activity [187]. In addition, Vpr-induced transcription from HIV-LTR and the Vpr-induced cell cycle abnormality can be inhibited by the naturally derived anti-HIV flavonoid quercetin [188]. Fumagillin, a natural yeast product and a known inhibitor of angiogenesis, not only reversed the growth inhibitory activity of Vpr in yeast and human cells, but also inhibited Vpr-dependent viral gene expression upon infection of human macrophages [189]. Other studies, which focused on the interaction of Vpr with the GR, described the anti-Vpr activity of mifepristone (RU486) and also neutralizing antibodies to Vpr [81,190,191]. Despite their high cytotoxicity, two PTPC inhibitors, cyclosporin A and bongkrekic acid, can specifically block Vpr-induced mitochondrial damage [83]. It was also reported that di-tryptophan-containing peptides inhibit HIV-1 Vpr-mediated apoptosis and G2 arrest in HIV-1-producing CD4^+^ T cell lines by interacting with Vpr and interfering with critical protein interactions [192]. Hematoxylin blocks virus replication by targeting the nuclear import of HIV-1 promoted by Vpr protein but does not block Vpr-induced G2 cell cycle arrest [193]. Moreover, a stable hematoxylin derivative strongly binds to the third α-helix of Vpr and inhibits HIV-1 replication in macrophages [194]. Screening of a chemical compound array identified SIP-1 (spirooxindole), which binds to Vpr and displays anti-HIV activity in macrophages by a yet unknown mechanism [195]. The 3-phenyl coumarin-based compound, vipirinin, was reported to inhibit the cell cycle arrest activity of Vpr in yeast and Vpr-dependent viral infection of human macrophages [196]. Recently, the caspase-1 inhibitor VX-765 was reported to improve neurobehavioral deficits observed in HIV-1 Vpr transgenic mice [197]. Finally, the use of compounds that minimize redox imbalance or increase intracellular ATP stores protects from Vpr-induced cytopathogenicity and consequently could prevent the neuropathogenesis associated with HIV-1 disease [107,108,198,199].

## 8. Conclusions and Perspectives

The current arsenal of antiretroviral agents is abundant but still insufficient. The development of novel targets to direct new antiretroviral drugs is necessary to combat residual problems of resistance, toxicity, and persistence of latent virus reservoirs in HAART regimens. The development of therapeutic drugs directed towards the multifaceted target Vpr might contribute to reduce these problems. Recent advances in the characterization of its interaction with host cells points to Vpr as having a changeable behavior depending on its multimerization status, protein concentration, and cell type infected. None of the Vpr-targeted inhibitors of HIV-1 infection are yet sufficiently potent to enter into clinical trials. The approach used in studies searching for Vpr-targeted antiretrovirals has focused on blocking one specific activity of Vpr during the virus life cycle, such as cell cycle arrest, apoptosis, or nuclear import. However, it is possible that a different approach will be required to efficiently block this multifaceted viral protein. The protection from energetic deficit and antioxidant production nullifying Vpr-induced pathogenesis represents a promising strategy in drug discovery of Vpr inhibitors. Several cellular models and transgenic animals are available for screening this Vpr-induced inhibition. Following the nuclear magnetic resonance (NMR) resolution of the Vpr structure, it is now possible to characterize the molecular interactions that Vpr might establish. Hence, a structure-based approach for drug design will also be very useful in the development of Vpr as a novel target for antiretroviral therapy. Along this line, molecular docking methodologies should be promoted in the rational design of new Vpr-targeted drugs.

## Figures and Tables

**Table 1:** Cellular proteins modulated by Vpr protein. (+): activation/increase; (−): inhibition/decrease.

Function	Cellular Protein	Effect	Reference
**Mitochondria/reactive oxigen species (ROS)**	ANT	+	[83]
	VDAC	+	[83]
	PMCA	−	[138]
	GLUD2	−	[94]
**Glucose metabolism**	HK1	+	[94]
**Pentose phosphate pathway**	G6PDH	+	[94]
**Glycolysis**	GAPDH	−	[94]
**Stress response**	ATR	+	[117]
**Proteasome**	DCAF1	+	[128]
**Transcription**	NFAT	+	[10]
	NF-ĸB	+/−	[79]/[39]
	C/EBP	+	[79]
	AP-1	+	[79]
	HIF-1α	+	[114]
	HDAC1	−	[122]
	GR	+	[141]
	SP1	+	[143]
**DNA metabolism**	SLX4	+	[66]
	HLTF	−	[59]
	UNG2	−	[148]
